# Jehovah’s Witnesses and Their Compliance with Regulations on Smoking and Blood Treatment

**DOI:** 10.3390/ijerph19010387

**Published:** 2021-12-30

**Authors:** Barbara Pavlikova, Jitse P. van Dijk

**Affiliations:** 1 Department of Labor Law and Social Welfare Law, Faculty of Law, Comenius University, 810 00 Bratislava, Slovakia; 2 Department of Community and Occupational Medicine, University Medical Center Groningen, University of Groningen, 9713 AV Groningen, The Netherlands; j.p.van.dijk@umcg.nl; 3 Graduate School Kosice Institute for Society and Health, Faculty of Medicine, P.J. Safarik University in Kosice, 040 01 Kosice, Slovakia; 4 Olomouc University Social Health Institute, Theological Faculty, Palacky University, 771 11 Olomouc, Czech Republic

**Keywords:** Jehovah’s Witnesses, compliance, smoking, blood, health laws

## Abstract

Jehovah’s Witnesses (JWs) are known as a religious group compliant with the national laws in the case of smoking, but not-compliant when it comes to blood treatment. Their beliefs prevent them from taking part in a blood transfusion, which is widely included in standard methods of a life-saving treatment. The aim of this study was to compare the behavior of JWs regarding their approach to blood treatment and to smoking in relation to legal regulations in the field of health. We measured JWs’ compliance with health laws regarding blood treatment and smoking (the Framework Convention on Tobacco Control—FCTC). We used the concept of a semi-autonomous social field by Moore and the theory of planned behavior developed by Ajzen. Our findings show that in the case of JWs, the group rules often prevail over state rules contained in generally-binding legislation. In the case of smoking, this means that they seem compliant to the FCTC and to their group rules. In the case of blood treatment, it seems that they are breaking the national rules, because of their group rules. Breaking the latter can result in exclusion from the JWs community. JWs are compliant with national laws as long as these are congruent with their own group rules. If this is not the case, the group influence is very strong and the fear of exclusion from the JW group is often greater than the potential negative health consequences in real life.

## 1. Introduction

The Jehovah’s Witnesses (JWs), founded in 1872, are an outgrowth of the International Bible Students Association. A member believed that Jehovah is the true God and that the Witnesses were his specially chosen followers. Later representatives continued and expanded the above-mentioned policies [1].

JWs base their moral code on the Bible and on the principles of sanctity of life, honesty and avoiding sexual misconduct, substance abuse (including smoking), superstitiousness, and angry behavior. The beliefs of JWs cover a number of traditional Christian views but also many that are unique [2]. JWs do not celebrate most holidays or events that people celebrate as non-religious holidays, such as birthdays, but they also do not celebrate religious holidays such as Christmas and Easter. JWs are extremely shunning to non-JWs or former members, including family members. JWs are advised not to join groups outside the JWs faith and are also discouraged from higher education [3].

JWs believe that they are living in the last days, and they look forward to the imminent establishment of God’s kingdom on earth, which will be headed by Christ and jointly administered by 144,000 human co-rulers. Their political teaching insists on a strict separation from secular government. Although they are generally law-abiding, believing that governments are established by God to maintain peace and order, they refuse on biblical grounds to be compliant with certain laws (e.g., even when compulsory, they do not vote or serve in the army). These practices have brought them under the scrutiny of government authorities. As a result, representatives and members of this religious group have often been persecuted and their faith condemned as a “cult” [1].

Approximately 8.7 million JWs live worldwide, with more than 120,000 congregations [4]. In Slovakia, there are 133 congregations and more than 11,000 ministers who teach the Bible [5]. The Census of 2011 recorded more than 17,000 JWs, which makes them the sixth largest religious society in Slovakia.

Teaching the Bible and missionary activities are the most important work of the religious group. JWs are trained from their youth to work as part-time missionaries for the whole of their lives. There is no distinction between professional clergy and lay people. All baptized members are considered ordained ministers. The Watch Tower Bible and Tract Society—one of a number of corporate entities that JWs use to accomplish their ministry—are controlled by the Governing Body (12 Members), which JWs believe is commissioned by Jehovah.

The Framework Convention on Tobacco Control (FCTC) is the first global public health treaty negotiated under the auspices of the World Health Organization (WHO). As a tool of so-called “soft law”, it provides an internationally coordinated response to combat the tobacco epidemic [6]. The FCTC was adopted in 2003 and came into force in 2005 [7]. Parties are committed to implementing the FCTC and its five main areas of interest, as identified by the creators of the Convention: effective tax and price policy; restriction of advertising and promoting of tobacco products; smoking bans; package warnings; and combating the illicit trade in tobacco [6,8]. However, due to its nature, the Convention does not contain any significant coercive tools, and its implementation is based on the willingness of the signatories to comply with its standards.

The concept of compliance in connection with legal rules brings an enriching point of view to the field of health care. It differs from adherence and persistence [9], and when we combine this concept with the factors influencing human behavior from “behind the scenes”, it enlightens the motivation of different groups of people (i.e., based on ethnicity, religion, age) to be compliant or non-compliant with the requirements contained in the health laws and not only with the recommendations of their physicians and pharmacists, which is one of the main characteristics.

In general, compliance means the willingness to act according to an order, set of rules or request [10]. Several opinions on compliance are in circulation [11,12]; in this case, we adhere to Von Stein [13], who understands compliance as the degree to which state (national, or lower) behavior conforms to what the agreement prescribes or proscribes. Non-compliance will then be defined as conflicting with such a prescription (expected behavior).

We focus on the behavior of a religious group represented by Jehovah’s Witnesses in regard to smoking and blood treatment in terms of legal regulations, with special attention paid to the FCTC and health laws regulating blood transfusions.

## 2. Materials and Methods

### 2.1. Sample

This study, based on the works of Moore (1973) [14] and Ajzen (1985) [15], has an analytical nature. JWs, an example of a religious group with very strict beliefs, have been selected as the object of the study. We focused on legal regulations contained in legislation, with special attention paid to the FCTC and other health laws.

We mainly worked with scientific articles published in relevant journals, such as *Law & Society Review*, *California Law Review* or *International Journal of Environmental Research and Public Health*, the official website of JWs [16], and legal sources of the World Health Organization [17]. No Ethics Committee approval was necessary for our documentary study, because we used information and data that are publicly available.

### 2.2. Measures

We used the concept of a semi-autonomous social field [14], the theory of planned behavior [15], and the FCTC as measurement tools.

JWs can be seen as an example of a semi-autonomous social field. Moore [14] explains the concept of a semi-autonomous field as an appropriate subject of study, because a selected field generating rules, customs and symbols internally, although it is still vulnerable to rules and decisions from the external environment, has rule-making capacity and is able to induce or coerce compliance. Autonomy and isolation are the core concepts of this theory [14]. Such minority social groups, which exist in every complex society, have something that Weber calls a “legal order” and are able to create their own rules. Group rules can sometimes appear to be superior to the national or state rules and can also override them, so the group acts as if no government exists [18]. The semi-autonomous field itself represents a form of “governance” [19].

Further, we measured the group beliefs with the application of the Ajzen’s Theory of Planned Behavior (TPB) [15]. We discussed the attitude, subjective norm, and perceived behavioral control. The relative importance of attitude, subjective norm, and perceived behavioral control in the prediction of the intention is expected to vary across behaviors and situations. People’s intention indicates how strongly they are willing to perform that behavior, which should be under their volitional control. Final success depends on intention and ability (behavioral control) [15,20]. Attitude is a psychological construct which is shaped by cognition (thought), values (beliefs) and affection (emotions) toward a particular object [21]. A subjective norm refers to the perceived social pressure to perform or not to perform a certain behavior. Norms are determined by the perceived social pressure from others on an individual. Perceived behavioral control refers to the perceived ease or difficulty of performing the behavior, and it reflects past experience as well as anticipated possibilities and/or obstacles [20].

Regarding the FCTC, we focused on two main areas of interest, as identified by the creators of the Convention: restriction of advertising and promoting of tobacco products and smoking bans.

### 2.3. Reporting

The aim of this study was to evaluate the behavior of JWs in regard to their approach to smoking in relation to legal regulations, with a special focus on the FCTC. We used JWs’ attitude towards smoking and to blood treatment as a comparative tool to search for what is crucial for the members of the community when it comes to the resulting behavior—whether to follow national or group rules.

We started with the description of JWs as a religious group. We approached their perceptions and beliefs, which can influence their behavior. We continued with the definition of the FCTC as the tool by which we evaluated the compliance of JWs in the area of health care norms. Further, we explained the concept of compliance.

In the Results, we present the approach of JWs to smoking and to blood treatment. We have chosen these two categories, because the views of the group members differ here, at least seemingly. On the basis of our findings, the analysis, and the confrontation with the other published works in the Discussion, we tried to draw conclusions regarding the compliance of JWs to legal norms in the field of health care.

## 3. Results

### 3.1. Approach of Jehovah’s Witnesses to Smoking

JWs consider smoking, together with alcoholism, drug abuse, and masturbation, as a weakness that should be treated appropriately, with the use of medical supervision, Bible study, prayer, and trusting congregational members for moral support [22].

There is no reference to the use of tobacco or other modern substances in the Bible [23]. However, JWs follow some principles related to the protection of their life and health, such as respect for life, love of your neighbor or the need to control their minds. This belief originates in the belief that God does not approve of unhealthy and unclean habits, such as smoking or drug use. The second argument against smoking is that the Bible states that to please God, one must obey the secular authorities. In many places, the law strictly prohibits the use of certain drugs [22]. Tobacco use was banned in 1973 and tobacco users are not to be accepted for JWs’ baptism. JWs can only be baptized if they discontinue smoking after “a reasonable period of time, such as six months” [24].

On the official website of the JWs, there is a questionnaire available, especially for young people, aimed at helping them to find the answers about smoking. At the end of the text, the reader finds recommendations on how to resist and how to quit [25]. This teaching can serve as a guideline for potential smokers.

### 3.2. Approach of Jehovah’s Witnesses to Blood Treatment

The view of blood treatment, especially blood transfusions, is mainly based on religion rather than on medicine [26]. Nearly all JWs refuse transfusions of whole blood (including preoperative autologous donation) and the primary blood components—red cells, platelets, white cells and unfractionated plasma. Many JWs accept the transfusion of derivatives of primary blood components, such as albumin solutions, cryoprecipitate, clotting factor concentrates, and immunoglobulins [27].

JWs cite the Old and New Testament as the Biblical basis for refusing transfusions. They believe that it is God’s will to “abstain from blood” (Genesis 9:4; Leviticus 17:10; Deuteronomy 12:23; Acts 15:28,29) [3] and that God has forbidden this. Accepting a blood transfusion willingly and without regret is seen as a sin. The JW concerned would no longer be regarded as one of JWs [28].

### 3.3. Compliance to the National Health Laws in the Field of Smoking and Medical Treatment

JWs see no reason to adhere, to be compliant, or to submit themselves to the authorities of the world, including governments, courts, and police, especially when laws go against their beliefs [3], because they believe that Satan controls the world and uses all of these entities as his tools. However, this religious group is very compliant to the national health laws, except in cases when the norms are against their religious beliefs.

As JWs see smoking as a “disfellowshipping offense,” members of the group are very compliant to the laws regulating tobacco use, including the FCTC. Based on the information above, it is obvious that they support a non-smoking environment organically, not out of loyalty to the State or the government, but because their faith asks them to do so. We can say that in this case group rules overrule national laws, but they pursue the same goal, so the result is the desired behavior.

In the case of medical treatment related to blood, more questions and problematic situations may arise. In many states, there is a legal possibility to refer to a previous wish of a patient in which the patient declared which type of medical treatment is acceptable to him or her and under which circumstances, when he or she does not have the capacity to do so. Many JWs carry a signed and witnessed advance directive card absolutely refusing blood and releasing doctors from any liability arising from this refusal [27].

## 4. Discussion

We studied the behavior of a religious group represented by JWs when it comes to smoking and blood treatment in relation to legal regulations. We found that in the case of smoking, JWs are compliant with the law, and in the case of blood treatment, they are not. JWs themselves consider the statement that they do not believe in medicine or medical treatment to be a myth [26]. The only difference in their view is that they are seeking doctors skilled in medical and surgical care without the use of blood.

### 4.1. Approach of Jehovah’s Witnesses to Smoking and to Blood Treatment

We found that JWs were compliant with legislation regarding smoking, but that they were not compliant with guidelines in the field of blood treatment. In this section, we discuss these findings. As the survey of Jakubowska et al. (2021) shows, there is a correlation between religious belief and attitude regarding drug abuse, including smoking. Other findings show that JWs prefer to follow group rules over medical recommendations when they are in conflict with one another [29].

From our point of view, JWs can best be seen as a semi-autonomous social group [14], as they are guided by their own group rules based on beliefs, and the way they live can be considered as a form of self-governance. JWs community members are separated from the non-JWs majority when it comes to decision-making processes, and they follow their own internal rules more than national (legal) rules when such rules go against those of the JWs community, and they are requested to do so under relatively strict sanctions.

Regarding Ajzen’s TPB, JWs’ attitude to smoking and medical treatment is led by the religious beliefs of the community, not by scientific data or possible health consequences. These beliefs overrule health laws, and if JWs are compliant with the law, it is not out of the fear of governmental penalties or of individual health consequences, but because the requests contained in the national legislation satisfy the community rules. Their subjective norms play a crucial role in the field of compliance. Individual JWs are motivated to comply with national rules or to not comply with them according to the expectations of the community. The JWs community expects its members first and foremost to be compliant with the group rules. If the rules satisfy the content of national health laws, then they are also compliant with them. If not, they will follow the rules of the community. Beliefs about sources and opportunities to behave in the expected way can also play a significant role when speaking about JWs’ compliance. The individual has many sources and support to be able to behave in the expected way.

### 4.2. Dilemmas

JWs refusal to accept blood makes some steps in medical treatment more dangerous. Doctors generally feel that respect for a JWs patient’s autonomy requires that this wish should be obeyed. Such a patient should have sound legal reasons for this too, as administering blood in the face of patient refusal may be against the law and could lead to criminal and/or civil proceedings [28]. Legally, such refusals are based on the constitutional grounds that a transfusion is an invasion of the right of integrity and a violation of the individual’s freedom of religious practice. When courts review these refusals, they focus on state interests, which outweigh the individual’s rights [30].

There have been cases where doctors have gone to court to get permission to give blood to children against the wishes of parents who are JWs. In 2000, the JWs changed the rules on blood transfusions such that the JWs Church would no longer take action against a JW who willingly and without regret underwent a blood transfusion. Some people wrongly interpreted the change as meaning that JWs could now accept blood. However, the actual change was just that the JWs Church would not take disciplinary action against that JW. If a JW is given a transfusion against their will, this is not regarded as a sin on the part of that individual. Children who are transfused against their parents’ wishes are not rejected or stigmatized in any way [28].

On the other hand, there is an argument that the development of no-blood treatment helps not only to JWs, but the whole community, which can benefit from the new possibilities of care. In many countries, any patient can now choose to avoid blood-transfusion risks, such as blood-borne diseases, immune-system reactions, and human errors [26].

### 4.3. Strengths and Limitations

JWs are a religious group with a very specific approach to many lifestyle questions. We looked at their beliefs through the lens of Moore and the TPB, which we consider to be the strength of this paper. The SASG and TPB theories can help us understand the behavior of different minorities (age, religious, political, ethnic…) better and adjust public policies as well as medical care in a way that helps achieve higher compliance with national health laws.

### 4.4. Implications

Further research in the field of providing medical treatment can be enriched by acquiring more hard data on the numbers of JWs who smoke and who refuse blood treatment, especially in the case of emergencies and life threatening events. In general, the point of view of medical staff and their experience with this religious group can be interesting, as well as interviews with the community members themselves about their approach to smoking and cessation. As there are new forms of tobacco products, such as e-cigarettes and vaporizers, research on the attitude regarding these new ways of using tobacco can be also conducted. Personal interviews could help explain the group beliefs more deeply and add more information.

## 5. Conclusions

JWs are compliant to legislation in the field of smoking and are non-compliant to legislation forcing blood treatment. We found that the reason for which they are or are not compliant to national and international legal regulations depends on whether the legal rules are in line with the religious beliefs of this group. If they are, JWs are compliant. If they are not, JWs behave according to the group rules.
